# Supplementary material for: Efficient Degradation of 2-Mercaptobenzothiazole and Other Emerging Pollutants by Recombinant Bacterial Dye-Decolorizing Peroxidases
Source: Biomolecules. 2021 Apr 29;11(5):656. doi: 10.3390/biom11050656 (PMC8146892; doi:10.3390/biom11050656)
Supplement: Supplementary file 1 [file biomolecules-11-00656-s001.zip › biomolecules-1158090-Supplementary Material.pdf]

Supplementary Materials

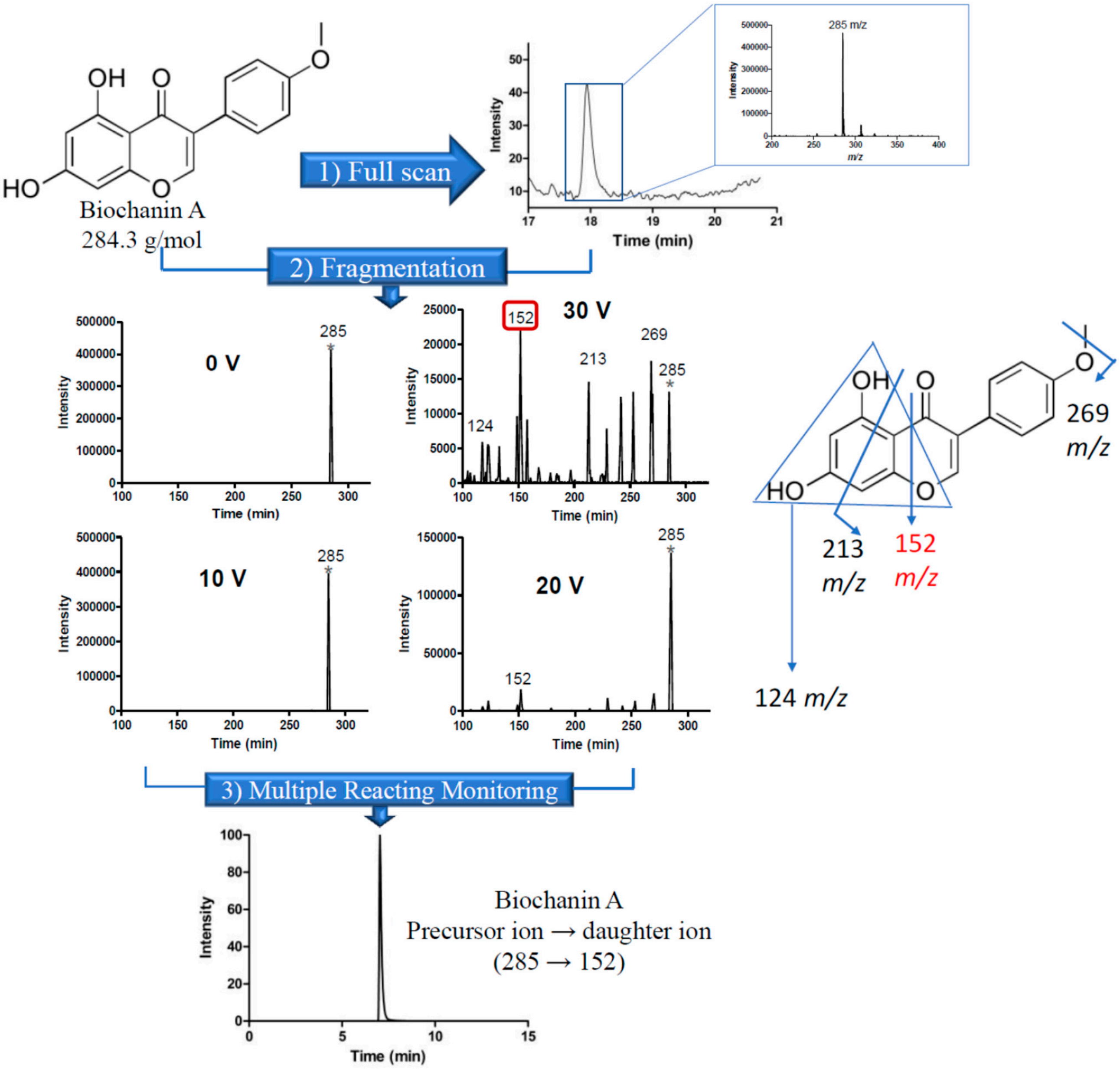

Figure S1: Schematic of the LC-MS MRM method development, using Biochanin A as an example.

Table S1: Summarized results showing % EP remaining upon HOBT-mediated treatment by rDyPs; (++++) = 0-25 % EP remaining, (+++) = 25-50 % EP remaining, (++) = 50- 75% EP remaining, (+) = 75-90 % EP remaining, (-) = no significant degradation. The greenish background indicates more degradation whereas the light reddish background shows less degradation.

DyPs / 31 EPs	YfeX	*Tfu*DyP	*Pf*DyP	*Tc*DyP	*Sc*DyP	*Svi*DyP	*Cbo*DyP
Acrylamide	++	++	++	+	-	+	-
MBT	-	+	-	-	-	++++	++++
Gemfibrozil	+	+	+++	++	-	-	-
Ibuprofen	+	+	+	-	-	+	-
Biochanin A	+	-	+	-	-	-	-
Cimetidine	+	-	+	-	-	-	-
Caffeic Acid	-	-	-	-	-	+++	++
Fluometuron	-	-	-	-	-	-	-
Venlafaxine	-	-	-	-	-	++	-
3-Methyl-2-benzothiazolinone	-	-	-	-	-	+	+
MCPA	-	-	-	-	+	+	++
Salicylic acid	-	-	-	-	-	+	+
Chloramphenicol	-	-	-	+	+	-	-
Lincomycin hydrochloride	-	-	-	-	-	+	-
DEET	-	-	-	-	-	-	+
Paracetamol	-	-	-	-	-	+	-
2-(Methylthio) benzothiazole	-	-	-	-	-	-	-
Sulfamethoxazole	-	+	+	-	-	-	-
Levetiracetam	-	-	+	-	-	-	-
Caffeine	-	-	-	-	-	-	-
Thiabendazole	-	-	-	-	-	-	-
Prometryn	-	-	-	-	-	+++	-
Phenytoin	++	+	-	-	-	-	-
Atenolol	-	-	-	-	-	-	-
Trimethoprim	-	-	-	-	-	-	-
Hydrochlorothiazide	-	-	-	-	-	-	-
Furosemide	-	-	+	-	-	-	-
Penicillin GK	+	-	-	-	-	-	++
Meloxicam	-	-	-	-	-	-	-
Roxithromycin	-	-	++	-	-	-	-
